# Effect of Synthesized Titanium Dioxide Nanofibers Weight Fraction on the Tribological Characteristics of Magnesium Nanocomposites Used in Biomedical Applications

**DOI:** 10.3390/nano13020294

**Published:** 2023-01-10

**Authors:** Ibrahim A. Alnaser, Hany S. Abdo, Mohamed S. Abdo, Mohamed Alkalla, Ahmed Fouly

**Affiliations:** 1 Mechanical Engineering Department, College of Engineering, King Saud University, Riyadh 11421, Saudi Arabia; 2 Mechanical Design and Materials Department, Faculty of Energy Engineering, Aswan University, Aswan 81521, Egypt; 3 Biomedical Engineering Department, Faculty of Engineering, Minia University, Minia 61519, Egypt; 4 Mechatronics Engineering and Intelligent Machines, School of Engineering, University of Central Lancashire, Preston PR1 2HE, UK; 5 Production Engineering and Mechanical Design Department, Mansoura University, Mansoura 35516, Egypt; 6 Department of Production Engineering and Mechanical Design, Faculty of Engineering, Minia University, Minia 61519, Egypt

**Keywords:** magnesium nanocomposite, artificial implants, titanium dioxide nanofibers, biomedical applications, electrospinning technique

## Abstract

Biomedical applications, such as artificial implants, are very significant for the disabled due to their usage in orthopedics. Nevertheless, available materials in such applications have insufficient mechanical and tribological properties. The current study investigated the mechanical and tribological properties of a biomedical metallic material, magnesium (Mg), after incorporating titanium dioxide nanofibers (TiO_2_) with different loading fractions. The TiO_2_ nanofibers were synthesized using the electrospinning technique. The ball-milling technique was utilized to ensure the homogenous distribution of TiO_2_ nanofibers inside the Mg matrix. Then, samples of the mixed powder with different loading fractions of TiO_2_ nanofibers, 0, 1, 3, 5, and 10 wt.%, were fabricated using a high-frequency induction heat sintering technique. The physicomechanical and tribological properties of the produced Mg/TiO_2_ nanocomposites were evaluated experimentally. Results showed an enhancement in mechanical properties and wear resistance accompanied by an increase in the weight fraction of TiO_2_ nanofibers up to 5%. A finite element model was built to assess the load-carrying capacity of the Mg/TiO_2_ composite to estimate different contact stresses during the frictional process. The finite element results showed an agreement with the experimental results.

## 1. Introduction

Total hip-joint replacement surgery has been considered one of the most popular techniques that support the disabled in the last three decades. However, materials used in such joints are still the main deficiency of such implants to achieve reliable performance. In 1891, Dr. Themistocles performed the first hip-joint replacement operation [1]. He used the ivory to form the femoral head of the hip joint and fixed it with nickel-coated screws. By 1940, an American surgeon called Dr. Austin Moore used metallic implants instead of the original bone, and the femoral head was fabricated from cobalt–chrome alloy Vitallium [2]. Such hip-joint replacement surgeries proved their ability to relieve pain, restore hip-joint function, and enhance mobility by using the artificial prosthetic hip joint [3]. A compatible artificial implant replaces the damaged femur head or bone in such surgeries. The artificial implant could be fabricated of ceramics, polymers, and metals and sometimes a combination of the three materials can be utilized [4]. The artificial hip-joint components are usually in direct contact with human blood, tissues, bones, and other fluids of the body; consequently, the utilized hip joint materials should be biocompatible. Such biocompatibility shows how the implanted joint materials could interact with living human tissues. According to the literature, three main factors have a crucial role when selecting materials for artificial implants: biocompatibility and the material’s mechanical and tribological properties [5]. Therefore, the selected material should be non-toxic and have no inflammatory reactions or hypersensitive responses to the cells and tissues of the human body [6].

Recently, a lot of emphasis on developing new biomedical materials has been given to achieve reliable materials that could be used in biomedical applications [7,8]. Initially, hip joint replacement was used with elderly people; however, nowadays, the technique is used with adults and young people who may be emerged from accidents. Such young people are more active and have hard motions, resulting in implant loosening(fracture) or wear of the joint [9]. However, wear of the hip joint is considered the most severe problem, limiting the artificial hip joint lifespan [10]. Increasing the wear of the hip joint leads to more suffering for the patient [11]. Consequently, researchers tried to develop different types of composites that can withstand different loads, such as metal on metal, metal on polymer, polymer on polymer, ceramic on ceramic, and ceramic on polymer.

Metals are considered the first choice when trying to find a strong and reliable material for an application [12,13]. Recently, the utilization of metals has been radically increasing for biomedical applications especially orthopedic joints, such as hip and knee joints [14]. Furthermore, metals are used for temporary implants, such as plates, pins, and screws. For more than a century, magnesium was used in vitro and vivo as a biodegradable implants [15]. Magnesium is usually nominated in different applications where light weight is needed due to its low density and acceptable mechanical properties [16]. Researchers could prove that magnesium and magnesium composites have a high degree of biocompatibility compared to other metals. Consequently, magnesium and its composites are the most valuable and nominated materials over other metallic materials used in implants [17,18]. Umeda et al. [19] investigated the metallic response of a popular magnesium alloy (AZ91D) in vivo and vitro situations. They proposed the utilization of magnesium as a matrix in metal composites in orthopedic applications. However, they found that the corrosion rate of magnesium is high. Neat magnesium has poor mechanical properties and a high wear rate when used in the biological environment [20]. Consequently, much research focuses on enhancing the mechanical and tribological performance of magnesium and its alloys. The selection of suitable additives with an optimal weight fraction to develop new magnesium composites with satisfactory mechanical and tribological properties represent the main challenge.

Due to the advantages and enhancement in the properties of metal matrix composites, various metals and their alloys are used as base matrices [21]. Usually, the filler used in the composite is chosen according to the desired attributes for the application. Titanium oxide as ceramic nanofibers is considered one of the most attractive materials for different applications [22]. With the ongoing development, and due to titanium oxide nanofiber unique characteristics, its usage is spreading in various applications, such as catalysts, pigments, photocatalysts, and cosmetics [23]. It was proven that the existence of titanium oxide on the titanium surface for prostheses could improve biocompatibility with bone tissue and increase corrosion resistance in the physiological environment [24,25]. Due to such advantages of titanium oxide, researchers categorized it as a biomaterial and used it in various treatments. Furthermore, it was proven that the incorporation of titanium oxide nanofiber into a metal matrix (aluminum) could improve the metal matrix mechanical properties [26,27,28]. In addition, many researchers used titanium oxide as a filler for enhancing the tribological properties of materials used in biomedical applications [29,30,31].

The preparation of titanium oxide nanofiber is well-known; electrospinning is a well-known electrohydrodynamic technique that is used to form TiO_2_ nanofibers [32]. In 1934, the technique of extracting TiO_2_ nanofibers was proposed by Anton Formhals, and in his patent, he described the preparation of the artificial thread in detail [33]. The technique was initiated by applying a high electric field on a liquid droplet to transform it into nanomaterial in which two different nanostructures could be achieved [34]. The first structure is nanoparticles, and in this situation, the process is called electrospraying. The second structure is long fibers which is called the electrospinning process, famous in tissue engineering applications [35]. The electrospun fibers are collected using a metallic capillary under negative high voltage and are in a randomly oriented non-woven condition [36].

As mentioned in the literature, magnesium has many advantages to being used as a biomedical material in orthopedic implants, such as hip joints. However, the mechanical and tribological properties of magnesium represent the main shortage for its usage. Consequently, the main objective of the current study is to investigate the effect of reinforcing magnesium by titanium oxide nanofibers on magnesium composite load-carrying capacity. The titanium oxide nanofibers were produced using the electrospinning technique. Then, the TiO_2_ nanofibers were incorporated into a magnesium matrix with different weight fractions 0, 1, 3, 5, and 10 wt.%. The physical, mechanical, and tribological properties were evaluated for the produced composites under different normal loads. The microstructure of the produced fibers, Mg/TiO_2_ mixture and worn surfaces were evaluated using a scanning electron microscope. To evaluate the load-carrying capacity, a finite element model was constructed to evaluate the contact stresses during the frictional process.

## 2. Materials and Experimental Work

### 2.1. Materials and Samples Preparation

The magnesium powder was purchased from Merck, Darmstadt, Germany, with a purity of 99%. Polyacrylonitrile and Polyvinylpyrrolidone (PVP) were purchased from Sigma-Aldrich, USA, with a molecular weight of 150,000 and 1,300,000, respectively. Dimethylformamide, Titanium isopropoxide (C_12_H_28_O_4_Ti) were obtained from the same company in the USA. Ethanol, with a purity of 96%, and Acetic Acid, 99.7% purity, were purchased from Avonchem, and Qualikems in Macclesfield, England, respectively. All the previous chemicals and solvents were utilized without purification (as received).

**Figure 1:**
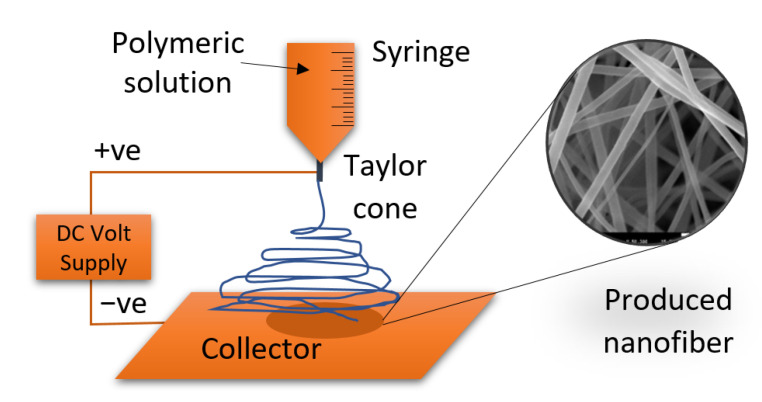
Schematic layout of the nanofiber electrospinning process.

To prepare the titanium dioxide nanofibers, the electrospinning technique was used. A sol-gel was prepared with 18 mL of acetic acid as a solution to gelation containing 60 g of ethanol and 3 g of PVP, and 9 g of Ti (IV)-isopropoxide. Then, the mixture was stirred for three hours at room temperature to produce a homogeneous viscous solution. A plastic syringe was loaded with the prepared solution where a blunt-ended stainless steel needle, 0.2 mm diameter, was connected to the output of the plastic syringe, as shown in Figure 1. The metallic needle was linked to a high-voltage source to apply 20 kV. The metallic needle has a tiny orifice which allows concentrated electric charge to be applied to a small droplet of the solution. Consequently, an electrically charged jet of the solution is produced. The solution-fed rate was adjusted utilizing a syringe pump to be 0.5 mL/h. The produced fibers were collected into a flat electrically grounded plate that was covered by an aluminum foil and placed 12 cm away from the needle tip. The nanofibers were collected into a cylindrical collector that rotates at 70 RPM to collect more nanofibers. According to the previous variables, the produced solution jet was evaporated and solidified before arriving at the cylindrical collector. All the previous process was conducted at room temperature. Eventually, calcination is conducted on the nanofiber to produce the titanium dioxide nanofibers [37,38,39]. Calcination is a heat treatment where the nanofiber is burned in an inert gas or air at a temperature higher than the thermal decomposition and lower than the melting point. In the current study, electrospun nanofibers were calcined in a tube furnace, CARBOLITE Type 3216CC, for 3 h at 800 °C with a heating rate of 15 °C/min. The tube furnace was fitted with an alumina tube with gastight fittings on each end. 

**Figure 2:**
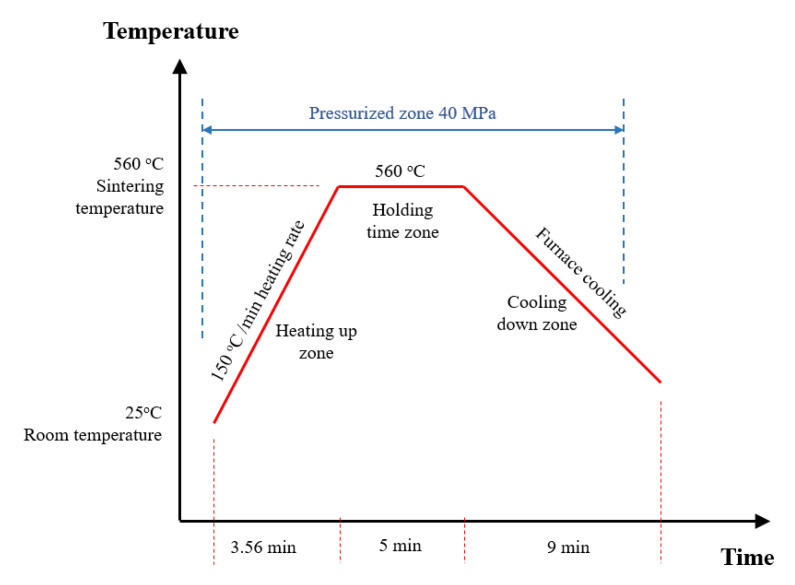
Consolidation process heat map.

Different techniques are used to ensure homogenous dispersion of the additive materials inside the matrix to prepare the composite samples. In the current study, the high energy ball-milling approach was utilized to blend magnesium powder with titanium dioxide nanofibers homogenously. Firstly, the magnesium powder was set for one hour into a stainless-steel jar and planetary ball mill (Desktop 220 V High Energy Vibratory Ball Mill with 80 mL Jar from Across International Company, Livingston, MT, USA) to de-agglomerate any coalesced powder. Secondly, the titanium dioxide nanofibers were added to the magnesium with different weight fractions 1, 3, 5, and 10 wt.% and mixed for half an hour to ensure homogenous dispersion of TiO_2_ nanofibers inside the Mg matrix. Thirdly, the nanocomposite mixture was inserted into the High-Frequency Induction Heat Sintering (HFIHS) process (HF Active Sinter System, ELTek CO., Gyeonggi-do, Republic of Korea) for consolidation. The Mg/TiO_2_ powder was loaded into a graphite die with an inner diameter of 10 mm and a height of 15 mm. The sintering temperature was adjustable and controllable using an optical pyrometer from Thermalert TX, Raytek GmbH, Berlin, Germany. During the sintering process, the mixture was exposed to a pressure of 40 MPA at a temperature of 560 °C heat rate of 150 °C/min for 5 min inside a vacuum chamber of 2 × 10^−3^ Torr to prohibit composite samples oxidation. Then, the Mg/TiO_2_ samples were left to cool until room temperature was reached. Figure 2 illustrates the heat map of the consolidation process.

### 2.2. Characterization and Testing

The morphology of the produced nanofibers and the Mg/TiO_2_ mixture were analyzed using a field emission scanning electron microscope (JEOL; JSM7600F). To investigate the physical properties of the produced MG/TiO_2_ composites, the density of each composition was evaluated using Archimedes’ principle. Each composite’s hardness and compression properties were estimated to evaluate the mechanical properties of the produced composites. After polishing each sample, the hardness was measured using a Vickers hardness tester (Buehler-micro-met 5114, Akashi Corporation, Osaka, Japan). The Vickers hardness test was conducted with a load of 500 g and a dwell time of 10 s. To ensure the hardness of each composition, the average hardness was estimated for five samples of each composite, and the standard error was calculated. The compression test was conducted according to ASTM: E9-89a [40] utilizing Instron 5582 Microtester (Instron, University Ave, Norwood, MA, USA). The strain rate during the test was set at 8 × 10^−5^ s^−1^. In addition, the compression test was performed three times for each composition.

**Figure 3:**
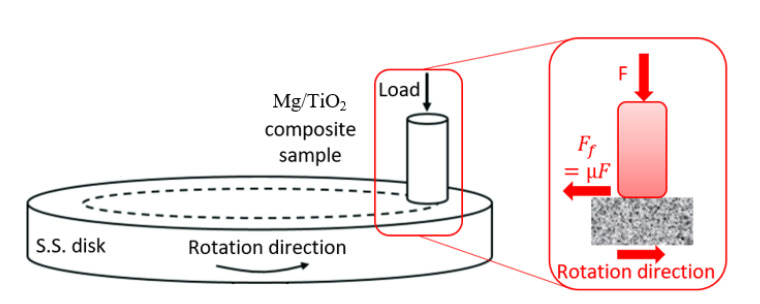
Schematic diagram for the wear test.

The tribological properties of the magnesium composites were evaluated using a pin on disk apparatus (universal tribometer Mod. UMT-2MT testing block sin T45815 Bruker-Nano Surfaces) in a dry rubbing condition based on ASTM G99-95 [41]. The Mg/TiO_2_ represented the pin of the apparatus, and the disk was made of stainless steel with a roughness of 13 μm. The Mg/TiO_2_ samples surface was polished before the experiment using fine-grade paper. Then, the sample surface was washed with acetone solution and dried using a high-pressure air jet to prevent any remaining contaminations before the experiment. The apparatus consists of two load cells, one for the normal force *F* and the other for the friction force *F_f_*. The friction coefficient *µ* was estimated by dividing the friction force by the normal force, as shown in the schematic diagram in Figure 3. To evaluate the specific wear rate, the Mg/TiO_2_ samples were weighed before and after each experiment. The wear test was performed five times for each Mg/TiO_2_ sample, and the average friction coefficient and specific wear rate were calculated. To investigate the wear mechanism, the worn surfaces were scanned using a field emission scanning electron microscope.

To evaluate the load-carrying capacity of the Mg composites, a finite element model was built using ANSYS software that represents the frictional process. The mechanical properties and friction coefficient of each composite were extracted from experiments and inserted into the ANSYS. The generated contact stresses were estimated on the surface of each composite. 

## 3. Results and Discussion

**Figure 4:**
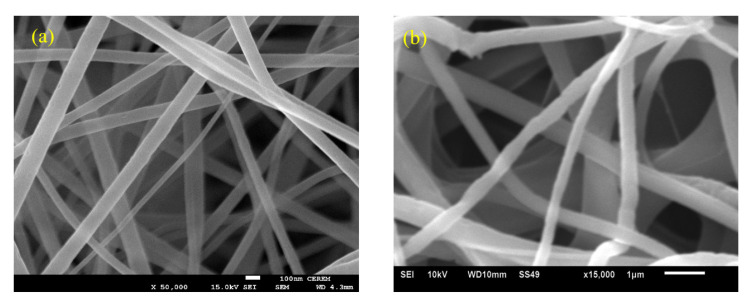
Titanium dioxide nanofibers (**a**) before calcination (reprinted with permission from [46]), and (**b**) after calcination.

**Figure 5:**
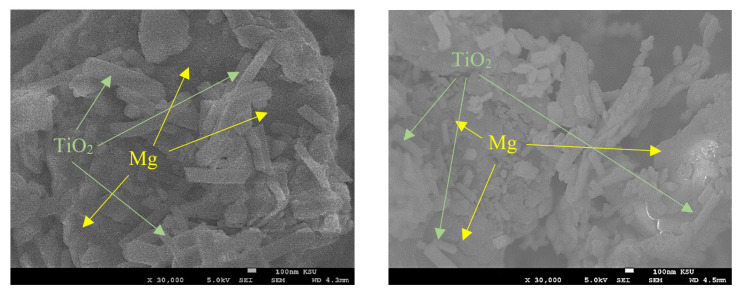
SEM images of ball-milled Mg/TiO_2_.

The morphologies of the titanium dioxide nanofibers as-spun and after calcination were evaluated using a field emission scanning electron microscope, as shown in Figure 4. It was reported before that the calcinated titanium dioxide fibers are usually in the anatase phase [42,43]. The FE-SEM images illustrate that the as-spun titanium dioxide nanofibers have an average diameter of 100 nm and have a uniform smooth surface with a random orientation. After calcination for 3 h at 800 °C, a significant change in the diameter of the nanofiber, around 0.407 µm. In addition, an insignificant distortion appears on the surface of the nanofibers, which may be occurred due to the crystallization of TiO_2_ and decomposition of PVP during the burning process [44,45]. After mixing magnesium with titanium dioxide nanofibers, the morphology of the mixed powder was scanned, as shown in Figure 5. SEM images show a homogenous distribution of the titanium dioxide nanofibers into the magnesium. 

As mentioned in the experimental work section, the magnesium nanocomposite samples were prepared with different weight fractions of titanium dioxide. The pure magnesium sample was denoted as Mg-TiO2-0. On the other hand, Mg-TiO2-1, Mg-TiO2-3, Mg-TiO2-5, and Mg-TiO2-10 represent magnesium with titanium dioxide nanofibers contents of 1, 3, 5, and 10 wt.%, respectively. The physical characteristics of any material could be represented by evaluating the density of such materials [47]. In the current study, the density of the Mg composites was measured, which depends on the densities of both magnesium and titanium dioxide nanofibers. Table 1 illustrates the change in the composite density with respect to the change in the weight fraction of the titanium dioxide nanofibers. The results showed a gradual increase in the Mg/TiO_2_ composites density with increasing TiO_2_ nanofibers weight fraction. Although the density increased due to the incorporation of TiO_2_ nanofibers into the magnesium, that increase is very slight, which preserves the advantage of magnesium being lightweight to be used in different applications.

The mechanical properties of the Mg/TiO_2_ composites were evaluated and listed in Table 1. The results show an enhancement in the hardness due to the incorporation of TiO_2_ nanofibers into the magnesium. The enhancement in the hardness indicates the homogenous distribution of the TiO_2_ nanofibers into the magnesium. As shown in the results, increasing TiO_2_ nanofibers led to an increase in the density of the composites, which might result in an enhancement in the hardness [48]. The maximum enhancement in the hardness was recorded for Mg-TiO2-10 with 74 Vickers hardness, 64.5% higher than pure magnesium with 45 Vickers hardness. By analyzing the compression test results, it is obvious that the incorporation of TiO_2_ nanofibers into the magnesium led to an enhancement in the ultimate and yield compressive strength. Table 1 shows that a continuous increase in both the ultimate and yield strength of the magnesium composite has occurred till TiO_2_ nanofiber weight fraction 5 wt.%. Increasing the weight fraction of TiO_2_ nanofiber to 10 wt.% led to a slight decrease in the strength of the magnesium composite. The slight decrease in the strength of the magnesium composite may be attributed to the high weight fraction of the titanium dioxide nanofibers that may result in some aggregations [49]. The maximum ultimate and yield strength were recorded for Mg-TiO2-5 by 281 MPa and 145 MPa, respectively. The ultimate and yield strength enhancement for Mg-TiO2-5 compared with Mg-TiO2-0 reached 12.5% and 45%, respectively. The enhancement in the composite strength indicates good interfacial bonding between the TiO_2_ nanofibers and the magnesium matrix clusters.

**Figure 6:**
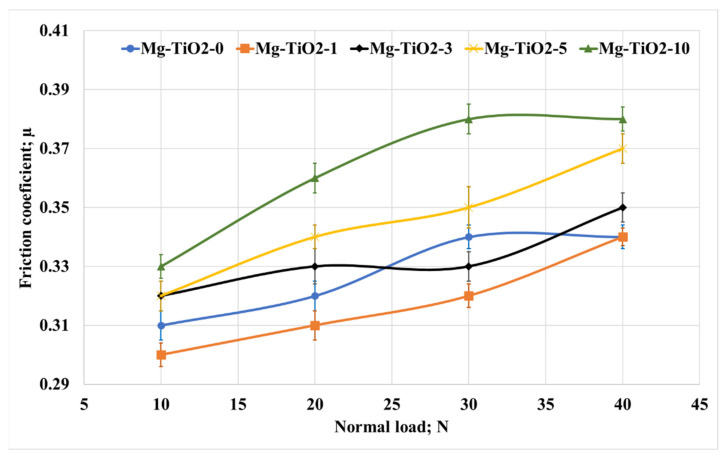
Friction coefficient of Mg/TiO_2_ nanocomposites under different normal loads, 900 m.

To evaluate the effect of titanium dioxide nanofibers loading fraction on the tribological properties of magnesium composites, the Mg/TiO_2_ nanocomposite samples were tested against a stainless-steel counterpart under different applied loads of 10–40 N, different sliding distances at a fixed sliding speed of 0.4 m/s and sliding distance of 900 m. Figure 6 illustrates the change in the friction coefficient for Mg/TiO_2_ nanocomposites under different applied loads. It is obvious that increasing TiO_2_ nanofibers led to an increase in the friction coefficient for different applied loads. The results reveal that the highest recorded friction coefficient was 0.38 for Mg-TiO2-10 at 40 N normal load, which is 18% higher than Mg-TiO2-0 (0.33). However, the friction coefficient was slightly decreased for Mg-TiO2-1 compared to Mg-TiO2-0, and it recorded the lowest friction coefficients among all Mg/TiO_2_ nanocomposites. Investigating the effect of the normal load, the results showed a gradual increase in the friction coefficient accompanied by the increase in the normal load. The increase in the normal load can result in an increase in the contact temperature in which the adhesion between the rubbed surfaces could increase and increase the friction coefficient [50,51].

**Figure 7:**
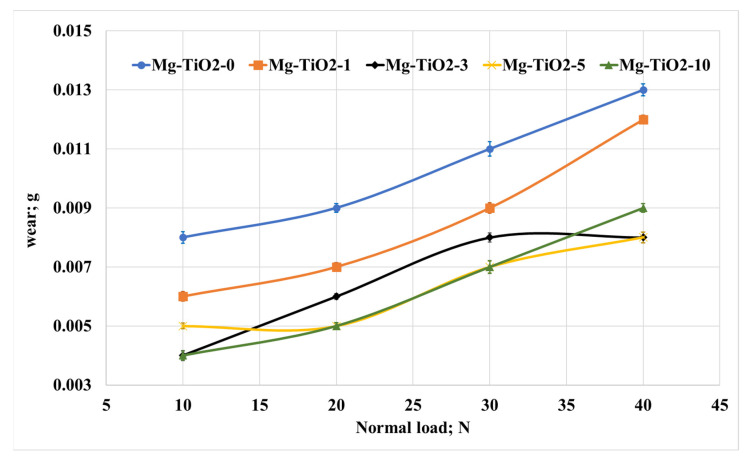
Wear of Mg/TiO_2_ nanocomposites under different normal loads, 900 m.

Regarding the effect of normal load and TiO_2_ nanofiber weight fraction on the wear of the Mg/TiO_2_ nanocomposites, Figure 7 illustrates the change in the weight loss of the Mg composite samples. Increasing the normal load led to a remarkable increase in weight loss for all composite samples. Such an increase in weight loss could be attributed to the generated heat during the frictional process, which depends on the kinetic energy during the test. This generated heat can weaken the surface of the composite sample and raise the shear resistance and increase the weight loss. In addition, increasing the contact temperature during the frictional process usually leads to a decline in the shear strength of the matrix, which also reflects the increase in weight loss [52].

On the other hand, Figure 7 shows that increasing the loading fraction of titanium dioxide nanofibers led to a significant decrease in weight loss. The results show that Mg-TiO2-5 nanocomposite recorded a 37.5%, 44.4%, 36.4%, and 38.5% decrease in weight loss compared with Mg-TiO2-0 at 10, 20, 30, and 40 N, respectively. The enhancement in the wear resistance could be attributed to the enhancement in the hardness and strength accompanied by the incorporation of TiO_2_ nanofibers into the Mg matrix.

**Figure 8:**
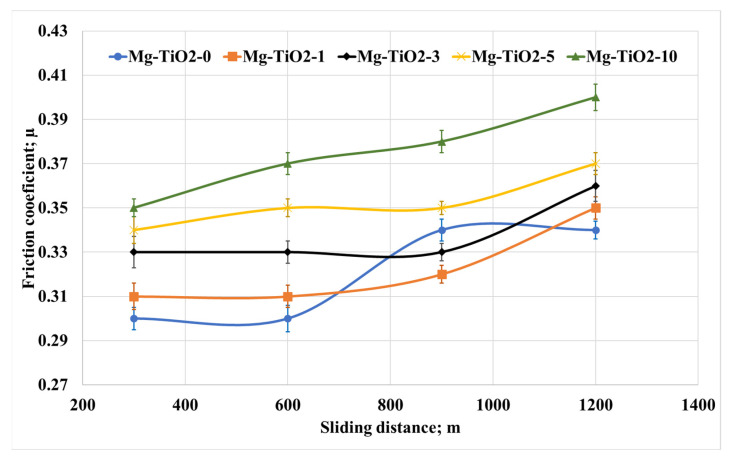
Friction coefficient of Mg/TiO_2_ nanocomposites under different sliding distances, 30 N.

**Figure 9:**
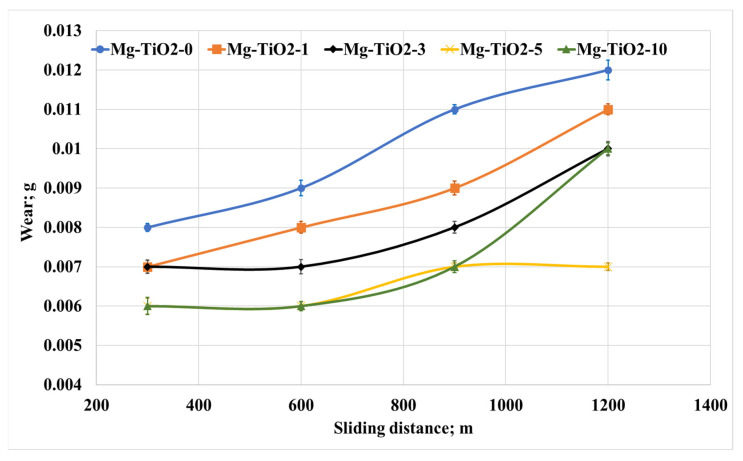
Wear of Mg/TiO_2_ nanocomposites under different sliding distances, 30 N.

As most components used in the frictional process work for a long time, the change in the tribological properties of Mg/TiO_2_ nanocomposites was evaluated with the change of the sliding distances. The friction tests were conducted under a normal load of 30 N for different sliding distances of 300, 600, 900, and 1200 m. The average friction coefficient and weight loss were measured and recorded, as shown in Figure 8 and Figure 9, respectively. Figure 8 shows an increase in the friction coefficient accompanied by an increase in the sliding distance. However, the tribological performance for all the composites remains the same, where Mg-TiO2-10 records the highest friction coefficient among other nanocomposites. As mentioned before, heat is generated due to the rubbing of the contact surface. Increasing the sliding distance increases the generated heat in which the adhesion force between the contact surfaces increases, leading to an increase in the friction coefficient. On the other hand, Figure 9 illustrates the weight loss due to the change in the sliding distance. It is obvious that Mg-TiO2-5 and Mg-TiO2-10 still have the lowest weight loss among other composites. However, a slight increase in weight loss occurred due to the increase in the sliding distances. For Mg-TiO2-5, the weight loss due to the increase in the sliding distance from 300 m to 1200 m is not exceeding 15%. The tribological results prove that incorporating TiO_2_ nanofibers into the Mg matrix could enhance wear resistance. These results may be attributed to the enhancement achieved in the mechanical properties of the composite.

**Figure 10:**
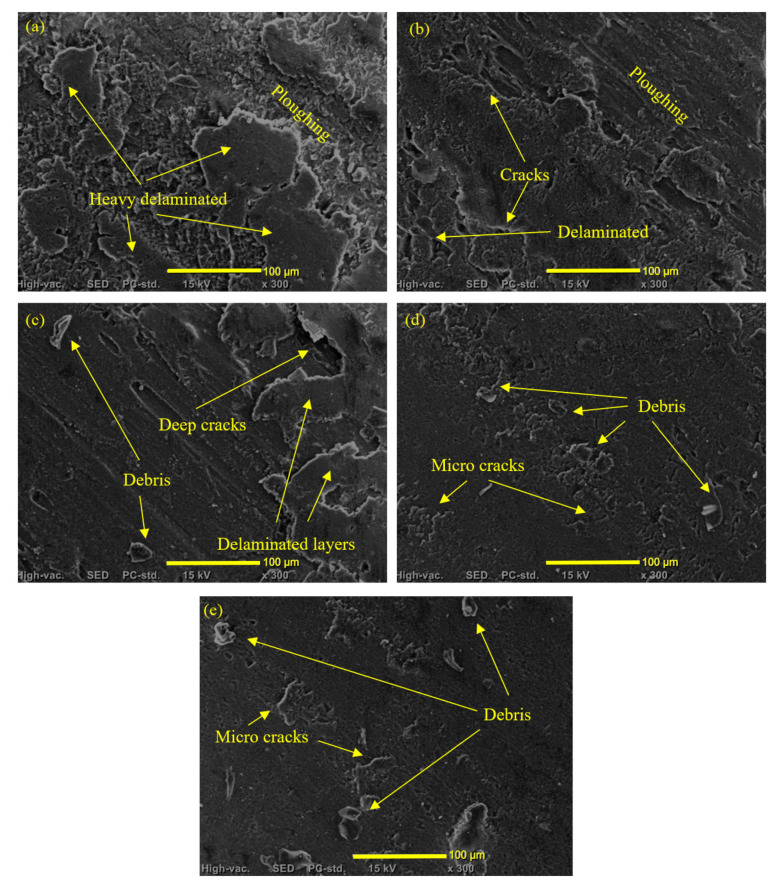
SEM of worn surfaces of (**a**) Mg-TiO2-0, (**b**) Mg-TiO2-1, (**c**) Mg-TiO2-3, (**d**) Mg-TiO2-5, and (**e**) Mg-TiO2-10.

To investigate the wear mechanisms that occurred during the frictional test of the Mg/TiO_2_ composites, the worn surfaces were analyzed using a scanning electron microscope, as shown in Figure 10. The wear mechanism analysis was conducted for testing parameters of 900 m sliding distance, 30 N normal load, and a fixed speed of 0.4 m/s. Before scanning the worn surfaces, a coating layer of platinum is used to enhance the conductivity of the sample. Figure 10a illustrates ploughing along the Mg-TiO2-0 sample in the direction of the frictional process. Cracks and delamination layers accompany the ploughed surfaces. By evaluating the worn surface of the pure magnesium, it is obvious that there is a combination between the abrasive wear mechanism and delamination wear mechanism—incorporation of TiO_2_ nanofibers up to 3 wt.%, Figure 10b,c, the ploughing and delamination decreased, and the cracks became more obvious. The decrease in the Mg-TiO2-1 and Mg-TiO2-3 surface deterioration could be attributed to the enhancement in the mechanical properties of the composite due to the addition of the TiO_2_ nanofibers. Such surface cracks show that the wear mechanism transfers from a delamination mechanism to a fatigue-delamination wear mechanism. Increasing the TiO_2_ nanofibers weight fraction to 5 wt.% enhanced the surface hardness and the composite’s bonding strength, improving the surface wear resistance, and reflecting smooth surface, as shown in Figure 10d with the existence of some microcracks and debris. In Figure 10d, The worn surface of Mg-TiO2-10 shows some micro-cracks and debris besides some voids that interpret the agglomeration that occurred due to the increase of TiO_2_ nanofibers weight fraction to 10%. Such agglomerated nanofibers were simply dragged away during the frictional test, and therefore, much wear debris appeared on the worn surface.

**Figure 11:**
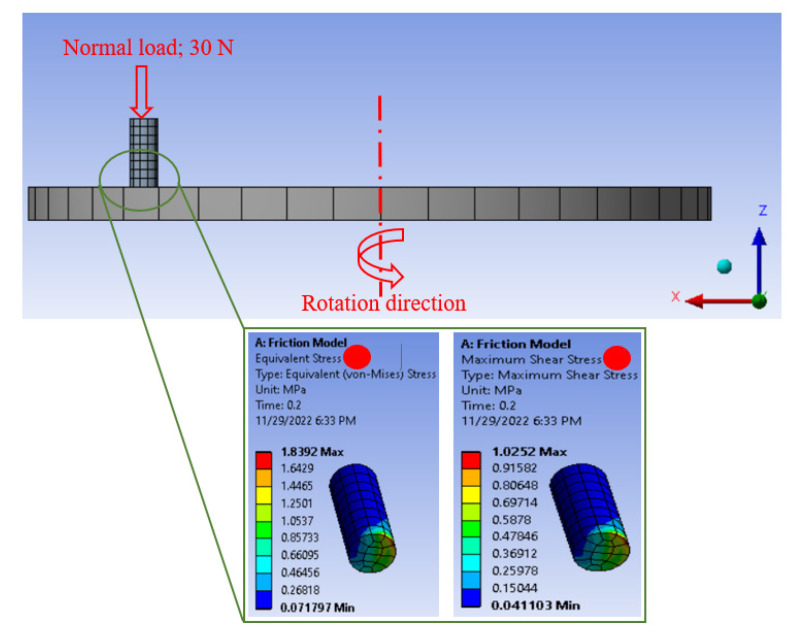
FEM of the friction process and the distributed contact stresses on Mg/TiO_2_ composite surface.

The contact stresses on the rubbing surfaces during the frictional process could indicate the load-carrying capacity of the tested composite. Kuminek et al. [53] proved that the load-carrying capacity increases as the contact stresses decrease during the rubbing process. Consequently, in the current study, a finite element model was built using ANSYS software representing the tribology test, as shown in Figure 11.

The disk in Figure 11 was defined as stainless steel, and the pin was defined as Mg/TiO_2_ composites where the composite properties were fed to the software based on the experimental results. Furthermore, the friction coefficient between the Mg/TiO_2_ composite pin and the stainless steel was defined based on the measured friction coefficient. An automatic mesh in the software was used to mesh the 3D model, and the element took the shapes of tetrahedron and hexahedron. The number of elements for the pin and the disk is 112 and 214, respectively. In addition, the node numbers for the pin and the disk are 637 and 1623, respectively. The Mg/TiO_2_ composite sample was subjected to a 30 N normal load in the z-direction; however, it was fixed in the other two directions.

**Figure 12:**
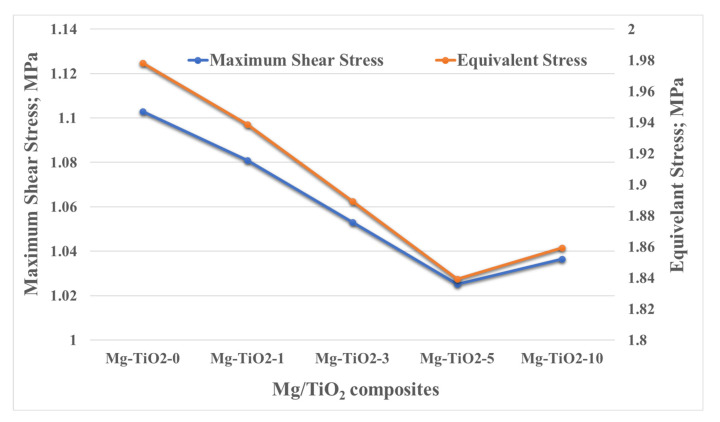
Resultant contact stresses on Mg/TiO_2_ composite surfaces.

Figure 12 shows the equivalent and maximum shear stress change due to incorporating TiO_2_ nanofibers into the Mg matrix. For both types of stresses, the maximum stress appears on the edge of the Mg/TiO_2_ composites, which is the starting point of the frictional process. The incorporation of TiO_2_ nanofibers into the Mg matrix obviously led to a decrease in both the equivalent and maximum shear stresses. Such a decrease in the contact stresses during the frictional process indicates an enhancement in the load-carrying capacity. The enhancement in the load-carrying capacity is in line with the experimental results where mechanical and wear resistance enhancement were achieved.

## 4. Conclusions

In the current investigation, novel metal matrix composites are proposed that can be used in biomedical applications. The metallic material used in the current study is magnesium due to its unique properties as a biomedical material. A ceramic nanofiber of TiO_2_ nanofibers was synthesized using the electrospinning technique as a reinforcement material. To fabricate the Mg/TiO_2_ composite samples, the Mg and TiO_2_, with different weight fractions, were mixed using high-energy ball milling. Then, the mixed powder was converted into a bulk composite specimen using the powder metallurgy technique through a high-frequency induction heat sintering furnace. Results revealed that an enhancement in the hardness reached 64.5% for Mg-TiO2-10 compared with pure Mg. In addition, an improvement in both ultimate strength, yield strength, and wear resistance for Mg-TiO2-5 compared with Mg-TiO2-0 reached 12.5%, 45%, and 44,4%, respectively. Eventually, a finite element model was constructed to evaluate the load-carrying capacity. FEA results showed an enhancement in the load-carrying capacity, which agreed with the experimental results. In the future work, further biological studies will be applied to the proposed composite to investigate its biocompatibility.

## Figures and Tables

**Table 1:** The physical and mechanical properties of Mg/TiO_2_ composites.

Sample	Composition (wt.%)	Density (gm/cm^3^)	Vickers Hardness	Ultimate Compressive Strength (MPa)	Yield Compressive Strength (MPa)
Mg-TiO2-0	Mg + 0%TiO_2_	1.7 ± 0.1	45 ± 1.4	250 ± 1.3	100 ± 0.95
Mg-TiO2-1	Mg + 1%TiO_2_	1.71 ± 0.2	55.52 ± 1.1	259 ± 0.85	133 ± 0.73
Mg-TiO2-3	Mg + 3%TiO_2_	1.74 ± 0.15	63.95 ± 1.6	272 ± 1.7	142 ± 1.1
Mg-TiO2-5	Mg + 5%TiO_2_	1.76 ± 0.13	66.83 ± 0.9	281 ± 1.5	145 ± 1.22
Mg-TiO2-10	Mg + 10%TiO_2_	1.82 ± 0.1	74.02 ± 0.13	276 ± 1.4	136 ± 1.16

## Data Availability

The data presented in this study are available on request from the corresponding authors.
